# Effects of statin on circulating microRNAome and predicted function regulatory network in patients with unstable angina

**DOI:** 10.1186/s12920-015-0082-4

**Published:** 2015-03-13

**Authors:** Jingjin Li, Hong Chen, Jingyi Ren, Junxian Song, Feng Zhang, Jing Zhang, Chongyou Lee, Sufang Li, Qiang Geng, Chengfu Cao, Ning Xu

**Affiliations:** Department of Cardiology, Peking University People’s hospital, No. 11 Xizhimen South Street, Beijing, 100044 China; Department of Medicine, Karolinska Institutet, Stockholm, Sweden

**Keywords:** Unstable angina, Statin, MicroRNA, System biology, Regulatory network

## Abstract

**Background:**

Statin therapy plays a pivotal role in stabilizing the plaque for unstable angina (UA) patients although its mechanism(s) remains largely unexplored. Here we aim to identify microRNAs (miRNAs) mediating the protective effect of statins in UA patients.

**Methods:**

MiRNAs Array was carried out to compare the circulating whole blood miRNA profile of UA patients treated with (n = 10) and without statin (n = 10) and plasma miRNA profile UA patients treated with (n = 5) and without statin (n = 5). 22 whole blood miRNAs and 19 plasma miRNAs were found significantly upregulated in statin group. Targets of these miRNAs were predicted by algoritms: Targetscan, Miranda and Diana microT, then clustered according to functions and cell types by using the Database for Annotation, Visualization and Integrated Discovery (DAVID). To reveal the enriched function pathways in human atherosclerotic plaque, we analyzed microarray data from GEO database, Coronary atherosclerotic plaque (n = 80); macrophages in ruptured plaque (n = 11); carotid atheroma plaque (n = 64); advanced carotid atherosclerotic plaque (n = 29) using Reactome database. Integrated analysis indicated that statin induced miRNAs mainly regulate the signaling pathways of Rho GTPase and hemostasis in human atherosclerotic lesion. In vulnerable plaque, additional immune system signaling was also targeted.

**Results:**

The data showed target genes regulated by these statin induced miRNAs majorly expressed in i) plaque macrophage and platelet, where they were involved in hemostasis process; ii) in monocyte to regulate NGF apoptosis; iii) and in endothelial cell function in Rho GTPase pathway. Integrate analysis indicated that statin induced miRNAs mainly regulate the signaling pathways of Rho GTPase and hemostasis in human atherosclerotic lesion.

**Conclusions:**

Our study suggest that statin induces the expression of multiple miRNAs in the circulation of UA patient, which play important roles by regulating signal pathways critical for the pathogenesis of UA.

**Electronic supplementary material:**

The online version of this article (doi:10.1186/s12920-015-0082-4) contains supplementary material, which is available to authorized users.

## Background

Acute coronary syndrome is the main cause of mortality in the world. Statins are the major prescribed medication for hyperlipidemia, and are also used in the primary and secondary prevention of coronary artery disease. Its main pharmaceutic effect is to inhibit 3-hydroxy-3-methylglutaryl coenzyme A (HMG-CoA) reductase, which improves the prognosis of patients. Recently, several experimental and clinical evidences have indicated that statin can also function in cholesterol independent ways [1,2]. For example, it can exert beneficial effect by enhancing endothelial function, suppressing inflammation, improving plaque stability, reducing hemostasis etc. [3,4]. Molecular mechanisms behind the pleiotropic effects of statin, especially in multi-cells and in multi-signaling pathways need to be further investigated. Moreover the post-translation regulation and its functional network during the process remain enigmatic.

MiRNAs are a class of small non-coding RNAs, which can regulate genes via post-translation inhibition by incorporating into the RNA induced silencing complex (RISC) and binding to the 3’ untranslated region (3’UTR) of target mRNAs [5]. Recent studies have demonstrated that miRNAs play important roles in several cardiovascular disorders. miRNAs are also involved in the pathogenesis of plaque lesions, such as to inhibit stability of neointima formation and to decrease the size of plaque, and regulate neovasculartization [6-8].

Notably, statin may exert its effect though the downstream signaling pathway via regulating miRNA expression [5]. To study the miRNAs affected by statin treatment, as well as the related gene networks regulated by these miRNAs, we characterized the dynamic change of circulation miRNAome for the UA patients after using miRNA Taqman Low Density Array. Furthermore, bioinformatics analyses were preformed to predict gene targets for these differentially expressed miRNA and to systematically analyze potential function(s) of miRNAs during the development of atherosclerotic in UA patients.

## Methods

### Study population

Whole blood samples were collected from UA patients (n = 30) enrolled at Peking University People’s Hospital. The protocols were approved by the ethics review board of Peking University People’s Hospital. Informed consent was obtained from each participant. Diagnosis of UA was made according to the ACC/AHA 2007 guidelines for the management of patients with unstable angina/non-ST-Elevation myocardial infarction and the ACC/AHA/ACP-ASIM 1999 guidelines for the management of patients with chronic stable angina. The patients presenting elevated level of troponin I (TNI) and/or creatine kinase (CK-MB), a history of severe hepatic dysfunction, renal dysfunction, leukemia, leukopenia, thrombocytopenia, ongoing inflammatory and malignant diseases were excluded.

### Whole blood and plasma sample collection and RNA extraction

Whole blood samples were collected into PAXgene tubes (PreAnalytiX). Blood samples were centrifugation at 1,200 g for 10 minutes at 4°C, plasma were collected from the supernatant then transferred to RNase-free tubes. Samples were store at −80°C right after collection.. The RNA was isolated by using preanalytix kit (Qiagen & BD company). Briefly, the pellet centrifuged for 10 min at 3000 g, and resuspended in 350 μl buffer BM1. 40 μl proteinase K and Buffer BM2 were added and incubation continued for 10 min at 55°C. Then transferred to PAXgene Shredder spin column and centrifuged for 3 min at 15,000 g. Supernatant of flow-through was transferred to microcentrifuge tube, and 700 μl isopropanol was added. The supernatant was loaded on PAXgene RNA spin column to bind total RNA. The total RNA was washed with buffer BM3, and DNA was digested with 10 μl DNaseI. RNA was washed with buffer BM3 and buffer BM4, eluted with Buffer BR5, and finally stored at −70°C. Quality of the RNA samples was analyzed by thermo multiskan FC (Thermofisher scientific).

### MicroRNAs Taqman low density array

15 ng of total RNAs were reverse transcribed using the Taqman miRNA reverse transcription kit with Taqman miRNA Multiplex RT assays (human pool) (Applied Biosystems, Foster City, CA, USA). The pre-amplification reaction products were then analyzed using Human MicroRNA TaqMan Low Density Arrays (TLDA) version 3.0 (Applied Biosystems). MiRNA expressions were normalized to the expressions of mammal U6, which remained constant in all the samples among different patient groups. Relative quantification software DataAssist was used to calculate miRNAs expression difference with comparative Ct method To find consistently differentially expressed miRNAs, the data were subjected to significance analysis of microarrays (SAM). MiRNAs with q value <0.0001% were considered to be differentially expressed.

### Bioinformatic analyses

The bioinformatic work flow of the study is summarized in supplement Figure 1. miRNA tissue expression profile was retrieved from miRWalk common organ target tools http://www.umm.uni-heidelberg.de/apps/zmf/mirwalk/ [9].Target prediction of miRNAs was performed by using three algorithms, TargetScan, miRanda and Diana-microT [10-12].

**Figure 1:**
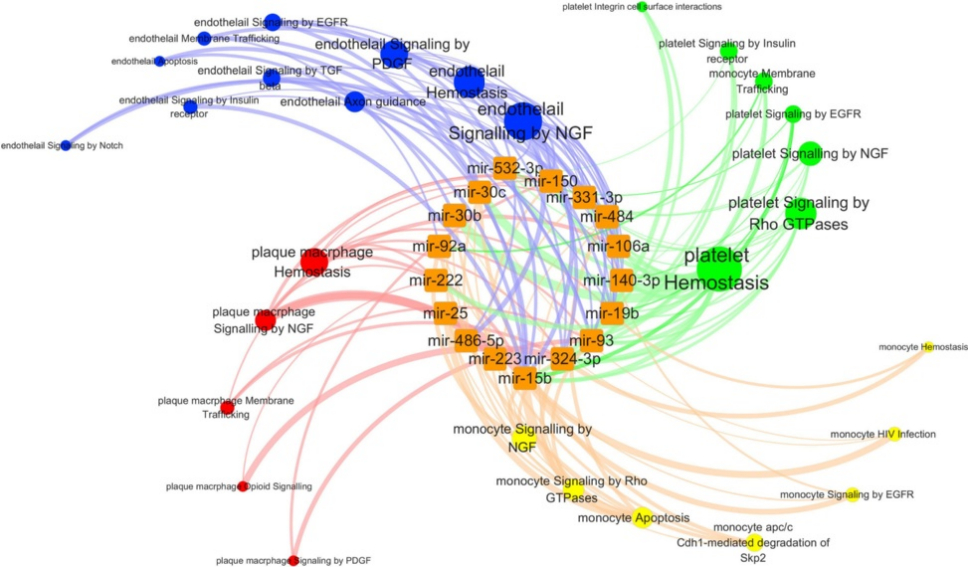
**MiRNA targeted signaling pathways in four different important cells including plaque macrophage, platelet, monocyte and endothelial cell.** Bioinformatic analysis was used to find significantly upregulated miRNAs target genes. Those target genes were clustered based on SAGE genie and categorized into four cell types. Each cell specific genes were then functionally clustered according to Reactome pathway. The two steps were performed in DAVID functional clustering tools and the figure was generated using CytoScape. The pathway which was targeted by only one miRNA was not displayed.

For these target genes, we classified them according to the cell type and their known functions by using Database for Annotation, Visualization and Integrated Discovery(DAVID) bioinformatics resources 6.7 http://david.abcc.ncifcrf.gov [13]. Clustering of target genes according to cell types were based on CGAP SAGE tissue-specific library [14]. The pathway enrichment analysis was done using reactome database [15]. To visualize the miRNAs targeted genes and cell specific functional pathways, we input network dataset into CytoScape version 3.0.0 beta 1 available at http://www.cytoscape.org [16].

To perform a integrated analysis of the miRNA involved in atherosclerotic lesions, we downloaded the following microarray data from the Gene Expression Omnibus (GEO) database (http://www.ncbi.nlm.nih.gov/geo/) [17]: microarray data of macrophages from human ruptured plaque and stable plaque (GSE41571), coronary atherosclerotic artery and internal mammary artery IMA(GSE40231) [18], early versus advanced carotid plaque (GSE28829) [19], distant macroscopically intact tissue versus atheroma plaque (GSE43292) [20]. The raw data were analyzed by SAM and the significant differentially expressed genes (FDR < 0.05) were selected for further analysis. DAVID was used to analysis these genes’ enriched pathway with Reactome database. The network of integrated miRNA targets and plaque lesions upregulated gene enriched pathway were illustrated by CytoScape version 3.0.0 beta 1.

## Results

### Statin treatment changed miRNAome in the circulation of UA patients

MicroRNA TLDA was performed to compare miRNA expression profiles in the whole blood samples from UA patients treated with (n = 10) or without statin (n = 10) and plasma samples from UA patients treated with (n = 5) or without statin (n = 5). For these two groups of patients, no significant differences were observed in baseline conditions, medical histories, medications and the results of laboratory testings (Table 1). The miRNAs expression profiles were significantly different between these two groups (FDR < 0.001%) and 22 whole blood miRNAs and 19 plasma miRNAs were found significantly upregulated in the statin treatment group compared with the control group (Table 2). The whole analysis flow was presented in diagram. (Additional file 1: Figure S1) MiRNAs profile of the statin treatment group and the control group were illustrated in heatmap (Additional file 2: Figure S2).

**Table 1:** **Characteristics of the study population treated with and without statin**

	**Whole blood**			**Plasma**		
	**Control(n = 10)**	**Stain(n = 10)**	**p value**	**Control(n = 5)**	**Stain(n = 5)**	**p value**
**General data**						
Sex, M/F	(4/6)	(5/5)	>0.99	(3/2)	(1/4)	0.524
Age(yrs)	59 ± 7.9	56 ± 7.6	0.394	59 ± 4.2	64 ± 9.9	0.343
SBP(mm Hg)	135 ± 15.1	131 ± 15.6	0.889	131 ± 12.1	128 ± 16.4	0.758
DBP(mm Hg)	80 ± 7.1	82 ± 12.4	0.591	78 ± 8.1	75 ± 11.2	0.669
**Medical history**						
Hypertension(%)	90(9/10)	80(8/10)	>0.99	60(3/5)	100(5/5)	0.444
Diabetes(%)	0(0/10)	0(0/10)	>0.99	20(1/5)	20(1/5)	>0.99
Hyperlipaemia(%)	30(3/10)	50(5/10)	0.649	60(3/5)	100(5/5)	0.444
Cerebral vascular disease (%)	0(0/10)	0(0/10)	>0.99	0(0/5)	0(0/5)	>0.99
Smoking(%)	20(2/10)	0(0/10)	0.474	40(2/5)	0(0/5)	0.444
**Laboratory test**						
Glucose(mmol/l)	5.4 ± 1.2	5.2 ± 1.4	0.796	4.8 ± 0.6	5.1 ± 0.6	0.526
LDL cholesterol (mmol/l)	2.5 ± 0.9	2.2 ± 0.9	0.577	2.6 ± 0.7	2.4 ± 0.4	0.605
HDl cholesterol (mmol/l)	1.0 ± 0.1	1.1 ± 0.3	0.314	1.2 ± 0.3	0.8 ± 0.1	0.034*
TC (mmol/l)	4.3 ± 0.9	4.0 ± 1.1	0.364	4.3 ± 0.5	3.9 ± 0.6	0.462
TG(mmol/l)	1.3 ± 0.6	1.2 ± 0.7	0.496	1.6 ± 0.2	1.6 ± 0.3	0.897
Creatinine(mg/dl)	60.7 ± 13.5	62.5 ± 18.8	0.781	76.2 ± 13.5	62.0 ± 20.2	0.781
alt(u/l)	28.9 ± 16.8	20.7 ± 9.2	0.211	21.6 ± 17.2	17.6 ± 5.3	0.616
ast(u/l)	23.4 ± 11.2	18.1 ± 4.27	0.224	18.8 ± 8.3	18.4 ± 2.3	0.897
**Medication**						
Aspirin(%)	30(3/10)	50(5/10)	0.649	40(2/5)	60(3/5)	>0.99
Clopidogrel(%)	30(3/10)	30(3/10)	>0.99	20(1/5)	60(3/5)	0.524
Calcium antagonists (%)	40(4/10)	20(2/10)	0.628	40(2/5)	20(1/5)	>0.99
ACEI/ARBs(%)	50(5/10)	20(2/10)	0.349	0(0/5)	20(1/5)	>0.99
β-Blockers(%)	20(2/10)	40(4/10)	0.628	40(2/5)	40(2/5)	>0.99

SBP, systolic blood pressure; DBP, diastolic blood pressure; LDL, low-density lipoprotein; HDL, high-density lipoprotein; TC, total cholesterol; TG, triglyceride; ALT, alanine aminotransferase; AST, aspartate aminotransferase; ACEI, angiotensin-converting enzyme inhibitor; and ARB, angiotensin receptor blocker. Comparisons between groups were performed with Student’s t test or Mann–Whitney U test for continuous variables and with the Fischer exact test or x^2^ test for categorical variables.

*p<0.05 versus control.

**Table 2:** **Significant differentially expressed miRNA profile in UA patients treated with or without statin**

**Whole blood**	**Score(d)**	**Fold change**	**q-value(%)**	**Plasma**	**Score(d)**	**Fold change**	**q-value(%)**
hsa-miR-191	3.9826	2.0182	<0.0001%	hsa-miR-21	4.0676	9.6700	<0.0001%
hsa-miR-92a	3.8634	2.6235	<0.0001%	hsa-miR-223	3.4159	8.6072	<0.0001%
hsa-miR-532-3p	3.3904	2.4110	<0.0001%	hsa-miR-106a	3.1782	5.9014	<0.0001%
hsa-miR-574-3p	3.1517	2.7277	<0.0001%	hsa-miR-17	3.0701	5.7191	<0.0001%
hsa-miR-30b	3.0671	2.2520	<0.0001%	hsa-miR-320	2.7697	7.9742	<0.0001%
hsa-miR-486-5p	3.0474	2.2180	<0.0001%	hsa-miR-24	2.5543	5.1630	<0.0001%
hsa-miR-223	3.0328	2.3800	<0.0001%	hsa-miR-146b-5p	2.4747	16.9902	<0.0001%
hsa-miR-30c	2.9640	2.1134	<0.0001%	hsa-miR-142-3p	2.4040	16.8645	<0.0001%
hsa-miR-484	2.9314	2.4529	<0.0001%	hsa-miR-19b	2.3810	7.1494	<0.0001%
hsa-miR-451	2.8041	3.1920	<0.0001%	hsa-miR-19a	2.2580	9.1338	<0.0001%
hsa-miR-331-3p	2.6506	1.8440	<0.0001%	hsa-miR-30a-5	2.1183	9.2122	<0.0001%
hsa-miR-25	2.6181	2.3039	<0.0001%	hsa-miR-222	2.0399	8.4189	<0.0001%
hsa-miR-222	2.5022	2.0910	<0.0001%	hsa-miR-126	2.0359	7.1429	<0.0001%
hsa-miR-652	2.4739	2.4337	<0.0001%	hsa-miR-451	1.9745	9.8249	<0.0001%
hsa-miR-19b	2.4250	2.0244	<0.0001%	hsa-miR-197	1.9738	2.7519	<0.0001%
hsa-miR-15b	2.2769	2.2809	<0.0001%	hsa-miR-92a	1.9470	6.4763	<0.0001%
hsa-miR-140-3p	2.2018	1.9416	<0.0001%	hsa-miR-20a	1.9312	6.8796	<0.0001%
hsa-miR-26a	2.1192	2.3774	<0.0001%	hsa-miR-29a	1.8389	4.4884	<0.0001%
hsa-miR-324-3p	2.1131	1.8923	<0.0001%	hsa-miR-146a	1.7932	7.7389	<0.0001%
hsa-miR-150	2.0946	1.7147	<0.0001%				
hsa-miR-93	2.0697	2.4706	<0.0001%				
hsa-miR-106a	2.0101	2.2727	<0.0001%				

22 whole blood miRNAs and 19 plasma miRNAs were significantly different between the statin treatment group and the control group.

### Identification of the cell origins of statin induced circulating miRNAs

To reveal the possible cell/tissue origin(s) for these differentially expressed circulating miRNAs, we searched miRWalk database for the organs which could express these microRNAs. We found that mir-15b, mir-150, mir-19b, mir-106a, mir-222, mir223, mir-26a, mir-30c, mir-451 and mir-92a were reported exist in monocyte. Mir-15b, mir-106a, mir-150, mir-191, mir-26a, mir-222, mir-223, mir-30b, mir-451, mir-92a and mir-93 can be expressed by macrophage. Mir-15b, mir-150, mir-223 mir-30b, mir-30c and mir-451 exist in platelet etc. In plasma, these MiRNAs were reported exited in monocyte mir-21, mir-24, mir-146b, mir-142-3p, mir-126, mir-20a, mir-146a, mir-222, mir-223, mir-19b, mir-451, mir-106a and mir-92a. mir-21, mir-17, mir-24, mir-146b, mir-142-3p, mir-19a, mir-126, mir-20a, mir-146a, mir-222, mir-223 and mir-106a in endothelial cell etc. (Additional file 3: Table S1). Thus, we can cluster these statin regulated miRNAs according to their cell origins.

### Signaling pathways regulated by statin induced whole blood miRNAs

We predicted target genes regulated by these statin induced miRNAs by using three public algorithms: TargetScan, miRanda, and Diana-microT (Additional file 4: Table S2). To analysis the mRNA targets calculated by the three tools, we input each of 22 miRNA targets into DAVID bioinformatic tools. In order to analysis the whole blood miRNA specific in different cells. Target genes were separated into groups using serial analysis of gene expression (SAGE) database based on different cell types. We focused on atherosclerosis related cells, including monocytes, plaque macrophage, platelet, and endothelial cell. The target gene enriched pathways were analyzed in each specific cell type and visualized using cytoscape. We found that the target genes regulated by statin induced miRNA were enriched in the following pathways: (1) pathways of hemostasis, NGF (Nerve Growth Factor), membrane trafficking, opioid and PDGF (platelet-derived growth factor),in plaque macrophages; (2) pathways of hemostasis, Rho GTPases, NGF, integrin cell surface interactions, insulin receptor, and EGFR (epidermal growth factor receptor) in platelet; (3) Monocyte related pathway of signaling by NGF, Rho GTPases, apoptosis etc.; (4) Endothelial cell related signaling by NGF, hemostasis, apoptosis, PDGF etc. In general, the hemostasis pathway is the major one in macrophage and platelet; the Rho GTPases pathway is mainly regulated in monocyte and platelet and the NGF pathway appear in all the cell types analyzed here.

### Chracterization of the pathways regulated by statin induced miRNA in atherosclerosis plaque

**Figure 2:**
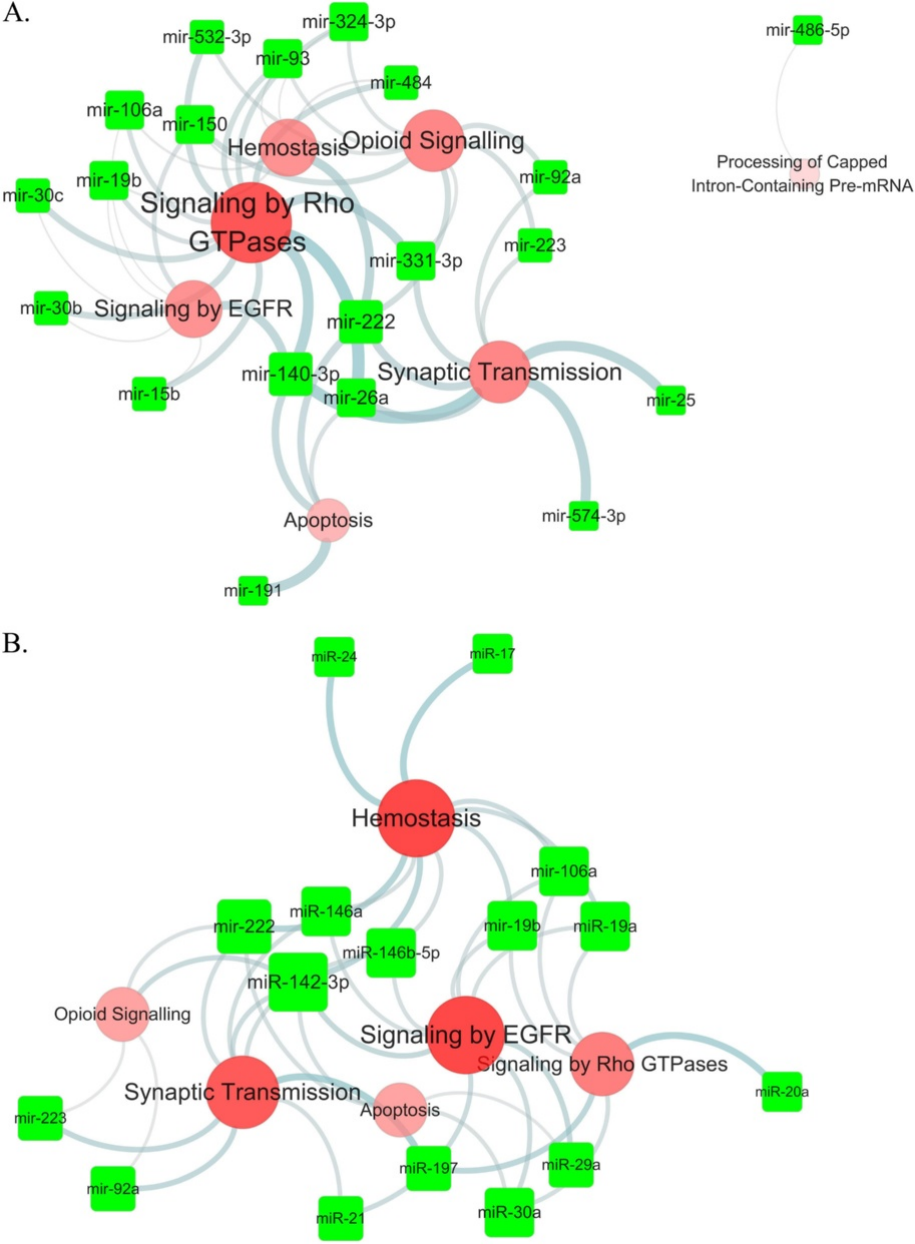
**MiRNA targeted the signaling pathways of upregulated genes in coronary atherosclerotic lesion.** The data which compared coronary atherosclerotic plaque and IMA (internal mammary artery) were obtained from GEO database. SAM was used to analyze the significantly differentially expressed genes (FDR<0.05). Those genes enriched pathways and miRNA target gene pathways were clustered by DAVID based on Reactome pathway. **A**: Whole blood miRNAs target pathways. **B**: plasma miRNAs target pathways. Illustration combined the miRNA target pathways which were also involved in the coronary atherosclerosis plaque.

**Figure 3:**
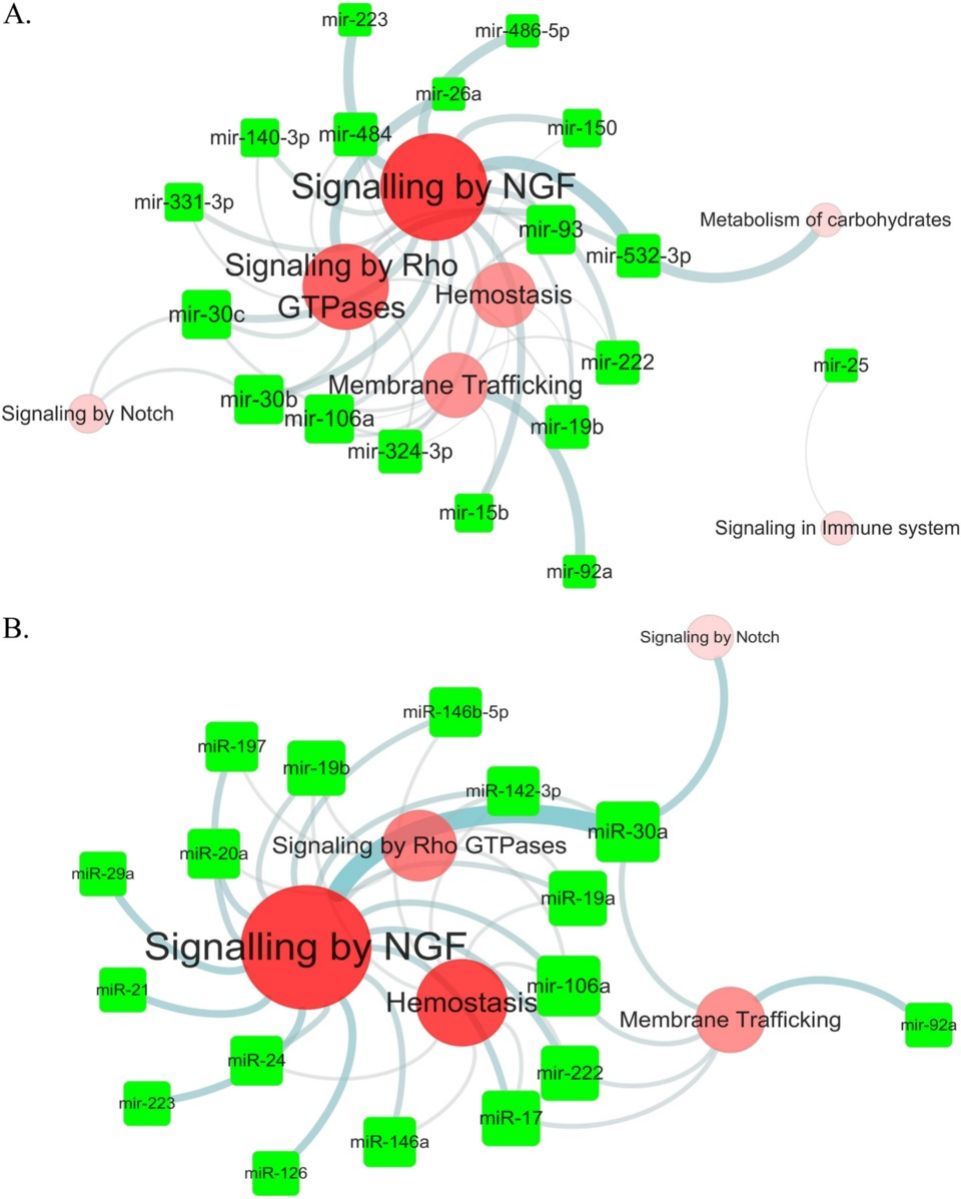
**MiRNA targeted the signaling pathways of upregulated genes from macrophages in ruptured atherosclerotic plaque.** The data which compared macrophage in ruptured atherosclerotic plaque with in stable plaque were obtained from GEO database. SAM was used to analyze the significantly differentially expressed genes (FDR<0.05). Those genes enriched pathways and miRNA target gene pathways were clustered by DAVID based on Reactome pathway. **A**: Whole blood miRNAs target pathways. **B**: plasma miRNAs target pathways. Illustration combined the miRNA target pathways which were also involved in the coronary atherosclerosis plaque.

To reveal the function of statin induced miRNA in atherosclerotic plaque, we analyzed four microarray datasets related to atherosclerosis from GEO database, and the alteration of pathways in the atherosclerotic lesion group were selected by enrich analysis of significant upregulated gene (Additional file 5: Table S3). The statin upregulated miRNAs in whole blood and plasma exert its pharmaceutical function by inhibit the upregulated gene in lesions. To characterize the putative functions of the miRNAs, we identified the genes that were upregulated in the disease group. Then we generated the putative function networks targeted by these statin induced miRNAs in each of the four datasets. In the two coronary artery datasets, the two networks indicated that both type miRNAs majorly targeted the pathways of Rho GTPases, hemostasis, EGFR, synaptic transmission and opioid signaling (Figure 2a, b). Other evidence from the dataset of resident macrophage of ruptured plaque is that both miRNAs target pathways major in NGF, Rho GTPases, hemostasis and membrane trafficking (Figure 3a, b). In the two carotid atherosclerosis datasets, we also have miRNA target involved pathway overlap with upregulated pathway in disease status. The NGF signaling pathway, hemostasis and Rho GTPase signaling also emerged in the carotid plaque (Additional file 6: Figure S3a, b). The tagets genes of statin induced miRNA target are also enriched in the hemostasis and Rho GTPase signaling in the advanced carotid plaque (Additional file 7: Figure S4a, b).

**Figure 4:**
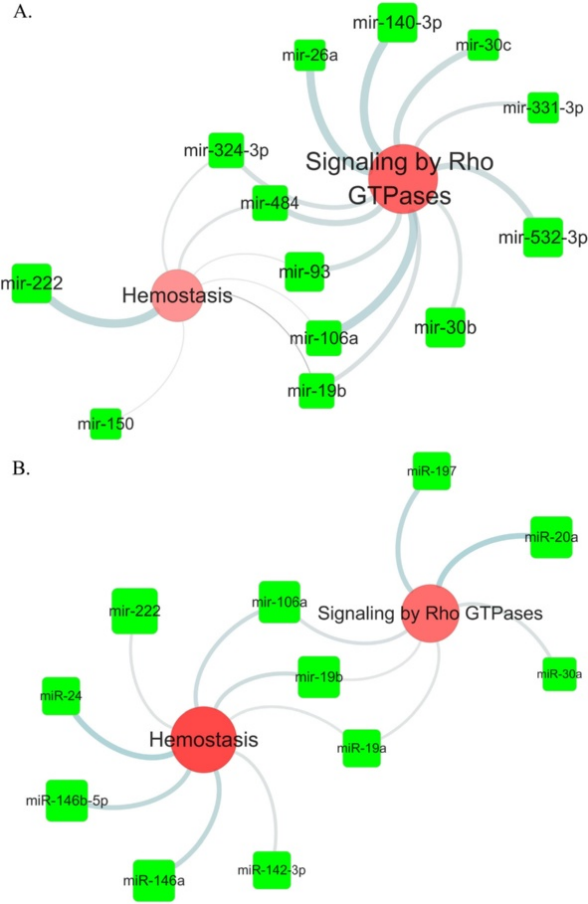
**Intersection network of the miRNA target pathway network.** The illustrated network was generated from the data of the miRNA target pathways in coronary atherosclerotic plaque, ruptured coronary plaque, carotid plaque and advanced carotid plaque. **A**. The network showed that the whole blood miRNA targeted Signaling by Rho GTPases and Hemostasis in the four atherosclerotic lesion. **B**. The network showed that the plasma miRNA targeted Signaling by Hemostasis and Rho GTPases in four atherosclerotic lesions.

Moreover, we combined the previous four miRNA target networks (fig 3,4 supplement fig 3,4). Results indicated that the whole blood miRNAs target Rho GTPase and hemostasis which were commonly enriched in four lesions (Figure 4a). The plasma miRNAs integrated network the hemostasis and Rho GTPase pathway (Figure 4b). These results indicated that statin upregulated miRNA targets functionally enriched in the two pathways in the disease status.

### Statin induced miRNAand their targets in Hemostasis and Rho GTPase pathways

**Figure 5:**
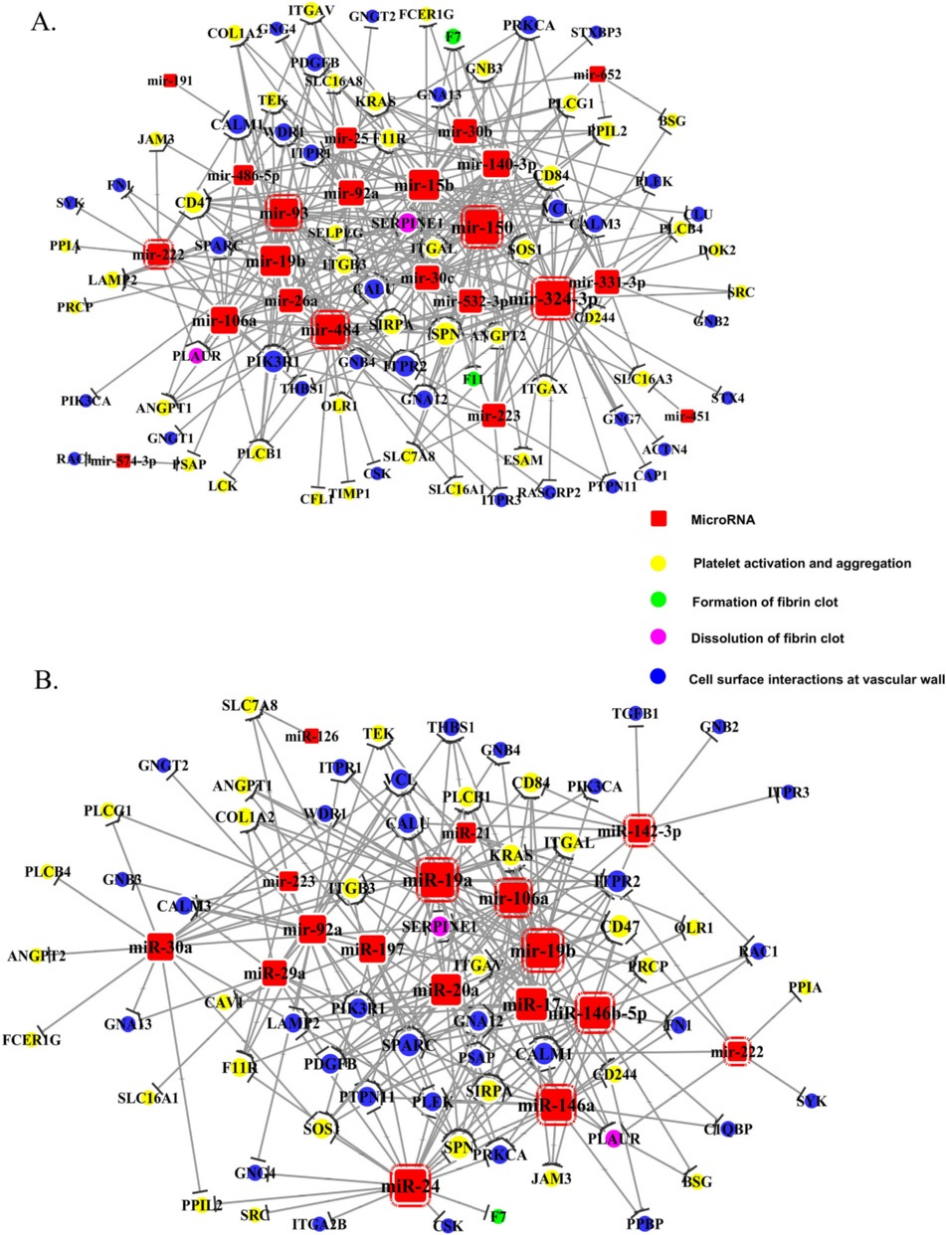
**MiRNA targeted upregulated genes related with hemostasis in plaque.** MiRNA targets were predicted by targetscan, Miranda and DIANAmT. The coronary plaque microarray data were analyzed by SAM and significantly differentially expressed genes were input into DAVID to select the hemostasis related genes. After integration of the miRNA targets genes and hemostasis related genes in the coronary plaque data, the miRNA target network was created to illustrate the mechanism how miRNAs were involved in the development of plaque. **A**: the network of whole blood miRNAs target genes. **B**: the network of plasma miRNAs target genes. The names of genes were official gene symbols. MicroRNAs with red border line are the miRNAs presents in the former network target the hemostasis. The yellow nodes are related with signaling of platelet activation and aggregation. The green nodes are related with formation of fibrin clot. The purple nodes are related with dissolution of fibrin clot. The blue nodes are related with cell surface interactions on the vascular wall.

**Figure 6:**
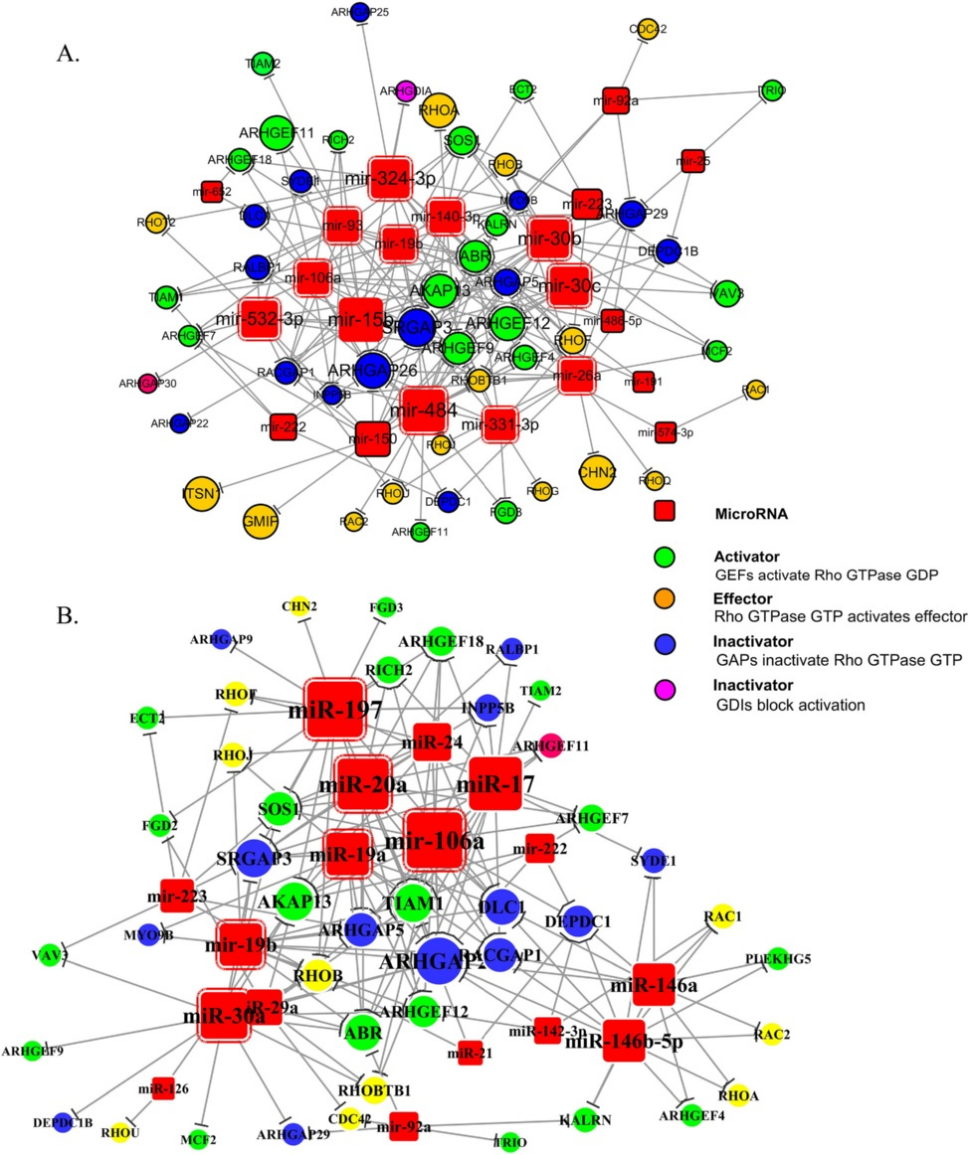
**MiRNA targeted upregulated genes of Rho GTPase signaling.** MiRNA target was predicted by targetscan, Miranda and DIANAmT. The ruptured plaque microarray data were analyzed by SAM, and significantly differentially expressed genes were input into DAVID to select the hemostasis related gene. The miRNA targets and Rho GTPase related genes were integrated. **A**: the network of whole blood miRNAs target genes. **B**: the network of plasma miRNAs target genes. The names of genes were official gene symbols. MicroRNAs with red border line are the miRNAs presents in the former network target the Rho GTPase pathway. The green node represents activation of Rho GTPase GDP by GEFS. The brown node represents activation of stream effector by Rho GTPase GTP. The blue node represents inactivation of Rho GTPase GTP by GAPs through hydrolysis. The purple node represents inactivation of Rho GTpase GDP by GDIs.

From above analysis, hemostatsis and Rho GTPase appeared to be two most important pathways targeted by these statin induced miRNAs in plaque, therefore we further analyze these two pathways. We made a network of miRNAs and their targets in hemostasis pathway (Figure 5a, b), showing that both whole blood and plasma miRNAs target genes were mainly involved in interactions of cell surfaces on the vascular wall, platelet activation signaling and aggregation. On the other hand, in Rho GTPase signaling pathway, whole blood and plasma miRNAs targeted both the activator GEFS and inactivator GAPs and GDIs of the Rho GTPase. Furthermore the downstream effectors of the pathway were also targeted by certain miRNAs (Figure 6a, b).

## Discussion

The benefits of Statins were caused by the inhibition of hydroxy-methyl-glutaryl-CoA (HMGCoA) reductase. Despite of the cholesterol lowering effect that ameliorated atherosclerotic plaque progression and reduced the adverse events in patients, accumulated evidence also showed that statins exerted pleiotropic effects [4,21-25]. In our study, the upregulated miRNAs downstream target in the specific cells most focused on the signaling pathway of NGF, Hemostasis, Rho GTPase and etc. As the circulating miRNAs could exert post-translation regulation in the target tissue, we preformed the miRNA and mRNA integrated analysis to describe statin induced miRNA function related to vascular disease. Previous studies have noticed the circulating microRNA have association with vascular disease [26,27]. We have analyzed the statin induced miRNAs which targeted the certain pathways, including the hemostasis, RhoGTPase and pathway in NGF rear reported in the atherosclerosis. Interestingly, our results are in accord with some previous findings such as hemostatsis and rho kinase pathway [28,29]. From the systematic aspect, our bioinformatic findings and functional predictions could explain the novel mechanism by which miRNAs regulated post-translational repression of certain biologic process.

Several clinical and basic studies suggested that statin had non-cholesterol-dependent effect on hemostasis. For example, The PROVE-IT (Pravastatin or Atorvastatin Evaluation and Infection Therapy) trial investigated high doses of statins in 4162 patients with acute coronary syndromes. The beneficial effect was independent of baseline LDL cholesterol and reached statistical significance [30]. In the JUPITER trial, rosuvastatin therapy was associated with decreased venous thromboembolism remarkably [31]. In a meta-analysis of the previous studies to investigate the efficacy of the immediate administration of statins in patients with acute coronary syndrome [32], the early beneficial effect was seen within acute phase (1 month). The significant reduction in clinical outcomes was associated with statin treatment, which played a potential role in antithrombosis. In mouse model, atorvastatin demonstrated an anticoagulation effect, which was induced by the reduction of platelet activation through upregulation of eNOS [33,34]. The abrupt withdrawal of statin abrogates the beneficial effect on endothelial function [4]. Furthermore, statins reduced tissue factor expression in macrophages and thereby inhibited the thrombotic interactions of the vascular wall [35]. We have noticed that the miRNA mostly inhibited the targets of hemostasis according to the bioinformatic analysis. It suggests that statin may act as an anticoagulant agent by increasing certain miRNA to inhibit coagulation. The specific aspect of miRNAs plays an important role in the process were mainly in signaling of the platelet activation and aggregation, and cell surface interactions on the vascular wall.

Meanwhile the association between Rho pathway and atherosclerotic lesions has long been studied. As the effectors of the small GTPase Rho, ROCKs have been shown to be upregulated in inflammatory arteriosclerotic lesions and to cause coronary vasospastic responses through inhibition of MLCP in both a porcine model of coronary artery spasm [36] and arteriosclerotic human arteries [37]. It has been suggested that ROCK induced NF-κB activation and subsequent T lymphocyte proliferation, which contributed to the development of early atherosclerosis [38]. Long-term inhibition of ROCKs caused a suppression of coronary arteriosclerosis and abrogation of coronary vasospastic activities in pig [39]. All the studies suggested that Rho GTPase signaling plays an important role in atherosclerosis, which was further demonstrated by our results that miRNA targeted the upregulated Rho GTPase pathway of coronary atherosclerotic plaque and ruptured plaque. Moreover, ROCK1-deficiency mice in bone-marrow-derived cells exhibited a phenotype of decreased plaques size in LDL-receptor deficient background [4]. ROCK1 is predominantly upregulated in macrophages that were activated and adherent to endothelial cells during atherosclerosis. ROCK1-deficient macrophages decreased chemotaxis, cholesterol uptake, and foam-cell formation, which resulted in regression of atherosclerotic lesions [40]. Our study suggested that circulating miRNA, which targeted Rho GTPase signaling, may also inhibit the plaque lesion. Thus, the anti-arteriosclerotic effect of statin may be partly due to the prevention of the activation of downstream Rho targets such as ROCKs [35]. Fluvastatin inhibited Rho in rat vascular smooth muscle cells, leading to the upregultion of iNOs expression [41]. Indeed, statins have been shown to inhibit Rho isoprenylation and ROCK activity. Statins can inhibit mevalonate synthesis, prevent membrane interaction of Rho, and suppress its subsequent activation of ROCKs [42]. Here we have provided a new aspect that miRNA reduced the RhoGTPase. MiRNA repressed target Rho GTPase signaling pathway involved in the plaque rupture. We showed that inhibition of Rho GTPase pathway appeared important in monocyte. Therefore, it is interesting to speculate whether some of the clinical benefits of statin therapy could be mediated by miRNA induced inhibition of the Rho GTPase pathways. So the miRNA induced inhibition of the effectors of the downstream may explain how statin have the effect on atherosclerosis.

On the other hand, the NGF signaling pathway and opioid pathway in our study are relatively new signaling pathways in atherosclerosis. NGF signaling is involved in the survival, differentiation and plasticity of neurons in the peripheral and central nervous system, and opioid pathway has powerful analgesic and sedative effects. These two signaling pathways exert important function in the signal transmission in neurons. However, studies, which investigated these two pathways in atherosclerosis were rear. NGF molecules decreased in the human coronary atherosclerotic plaque [43], and also reduced in the plasma of ACS patients [44]. The NGF signaling pathway, which was targeted by miRNA, may be also involved in the intervention of diseases. The pain of patients could be influenced by statin regulated miRNAs, which target in the opioid pathway. Further study is needed to unveil the potential effect of statin.

In summary, the combined data mining of miRNA and mRNA expression provided a putative mechanism to explain partial effect of statin that has been noticed.

## Conclusions

MiRNAs array indicated that statin induced 22 miRNAs in whole blood and 19 miRNAs in plasma. Bioinformatic analysis of the miRNAs signature revealed that statin influenced different pathways in the atherosclerosis related cells. Together with data from atherosclerosis lesions, miRNAs were also involved in the development of atherosclerotic lesion by targeting Hemosatsis and Rho GTPase pathway. All the results provided systematic evidence for regulatory effect of statin in unstable angina patients. Bioinformatic analysis was used to find significantly upregulated miRNAs target genes. Those target genes were clustered based on SAGE genie and categorized into four cell types. Each cell specific genes were then functionally clustered according to Reactome pathway. The two steps were performed in DAVID functional clustering tools and the figure was generated using CytoScape. The pathway which was targeted by only one miRNA was not displayed.

The data which compared coronary atherosclerotic plaque and IMA (internal mammary artery) were obtained from GEO database. SAM was used to analyze the significantly differentially expressed genes (FDR < 0.05). Those genes enriched pathways and miRNA target gene pathways were clustered by DAVID based on Reactome pathway. A: Whole blood miRNAs target pathways. B: plasma miRNAs target pathways. Illustration combined the miRNA target pathways which were also involved in the coronary atherosclerosis plaque.

The data which compared macrophage in ruptured atherosclerotic plaque with in stable plaque were obtained from GEO database. SAM was used to analyze the significantly differentially expressed genes (FDR < 0.05). Those genes enriched pathways and miRNA target gene pathways were clustered by DAVID based on Reactome pathway. A: Whole blood miRNAs target pathways. B: plasma miRNAs target pathways. Illustration combined the miRNA target pathways which were also involved in the coronary atherosclerosis plaque.

The illustrated network was generated from the data of the miRNA target pathways in coronary atherosclerotic plaque, ruptured coronary plaque, carotid plaque and advanced carotid plaque. A. The network showed that the whole blood miRNA targeted Signaling by Rho GTPases and Hemostasis in the four atherosclerotic lesion. B. The network showed that the plasma miRNA targeted Signaling by Hemostasis and Rho GTPases in four atherosclerotic lesions.

MiRNA targets were predicted by targetscan, Miranda and DIANAmT. The coronary plaque microarray data were analyzed by SAM and significantly differentially expressed genes were input into DAVID to select the hemostasis related genes. After integration of the miRNA targets genes and hemostasis related genes in the coronary plaque data, the miRNA target network was created to illustrate the mechanism how miRNAs were involved in the development of plaque. A: the network of whole blood miRNAs target genes. B: the network of plasma miRNAs target genes. The names of genes were official gene symbols. MicroRNAs with red border line are the miRNAs presents in the former network target the hemostasis. The yellow nodes are related with signaling of platelet activation and aggregation. The green nodes are related with formation of fibrin clot. The purple nodes are related with dissolution of fibrin clot. The blue nodes are related with cell surface interactions on the vascular wall.

MiRNA target was predicted by targetscan, Miranda and DIANAmT. The ruptured plaque microarray data were analyzed by SAM, and significantly differentially expressed genes were input into DAVID to select the hemostasis related gene. The miRNA targets and Rho GTPase related genes were integrated. A: the network of whole blood miRNAs target genes. B: the network of plasma miRNAs target genes. The names of genes were official gene symbols. MicroRNAs with red border line are the miRNAs presents in the former network target the Rho GTPase pathway. The green node represents activation of Rho GTPase GDP by GEFS. The brown node represents activation of stream effector by Rho GTPase GTP. The blue node represents inactivation of Rho GTPase GTP by GAPs through hydrolysis. The purple node represents inactivation of Rho GTpase GDP by GDIs.

## Additional files

Additional file 1: Figure S1. Study work flow diagram. Whole blood samples were collected from UA patients (n = 30) enrolled at Peking University People’s Hospital. TLDA, TaqMan Low Density Arrays; SAM, Significance Analysis of Microarrays; CGAP SAGE, Cancer Genome Anatomy Project Serial Analysis of Gene Expression; GEO, Gene expression Omnibus. Additional file 2:
**Profile of circulating miRNAs in non-statin group and statin group.** A: Whole blood differential miRNAs profiles. B: plasma miRNAs differential miRNAs profiles. Heatmap displays the levels of significantly differential expressed miRNAs. Color intensity represents the expression value in each row, bright red indicates the high expression level and the bright green indicates the low expression level. Additional file 3: Table S1. MiRNAs organ expression profile were generate from miRWalk database. The organ expression profiles of the miRNAs were based on published data. Additional file 4: Table S2. Enriched pathways of the MiRNAs’ target genes. Target genes list of significant differential MiRNAs predicted by TargetScan, miRanda, and Diana-microT. Additional file 5: Table S3. Enriched pathways of upregulated genes in atherosclerosis lesions. Additional file 6: Figure S3. MicroRNA target signaling pathway in the human cartoid plaque. The data which compared human carotid plaque and intact carotid tissue were obtained from GEO database. SAM was used to analyze the significantly differentially expressed genes (FDR < 0.05). Those genes enriched pathways and miRNA target gene pathways were clustered by DAVID based on Reactome pathway. A: Whole blood miRNAs target pathways. B: plasma miRNAs target pathways. Illustration combined the miRNA target pathways which were also involved in the coronary atherosclerosis plaque. Additional file 7: Figure S4. MicroRNA target signaling pathway in the human advance carotid plaque. The data which compared advance carotid plaque and early carotid plaque were obtained from GEO database. SAM was used to analyze the significantly differentially expressed genes (FDR < 0.05). Those genes enriched pathways and miRNA target gene pathways were clustered by DAVID based on Reactome pathway. A: Whole blood miRNAs target pathways. B: plasma miRNAs target pathways. Illustration combined the miRNA target pathways which were also involved in the coronary atherosclerosis plaque.
