# Supplementary figures and images for: Effects of statin on circulating microRNAome and predicted function regulatory network in patients with unstable angina
Source: BMC Med Genomics. 2015 Mar 13;8:12. doi: 10.1186/s12920-015-0082-4 (PMC4364658; doi:10.1186/s12920-015-0082-4)

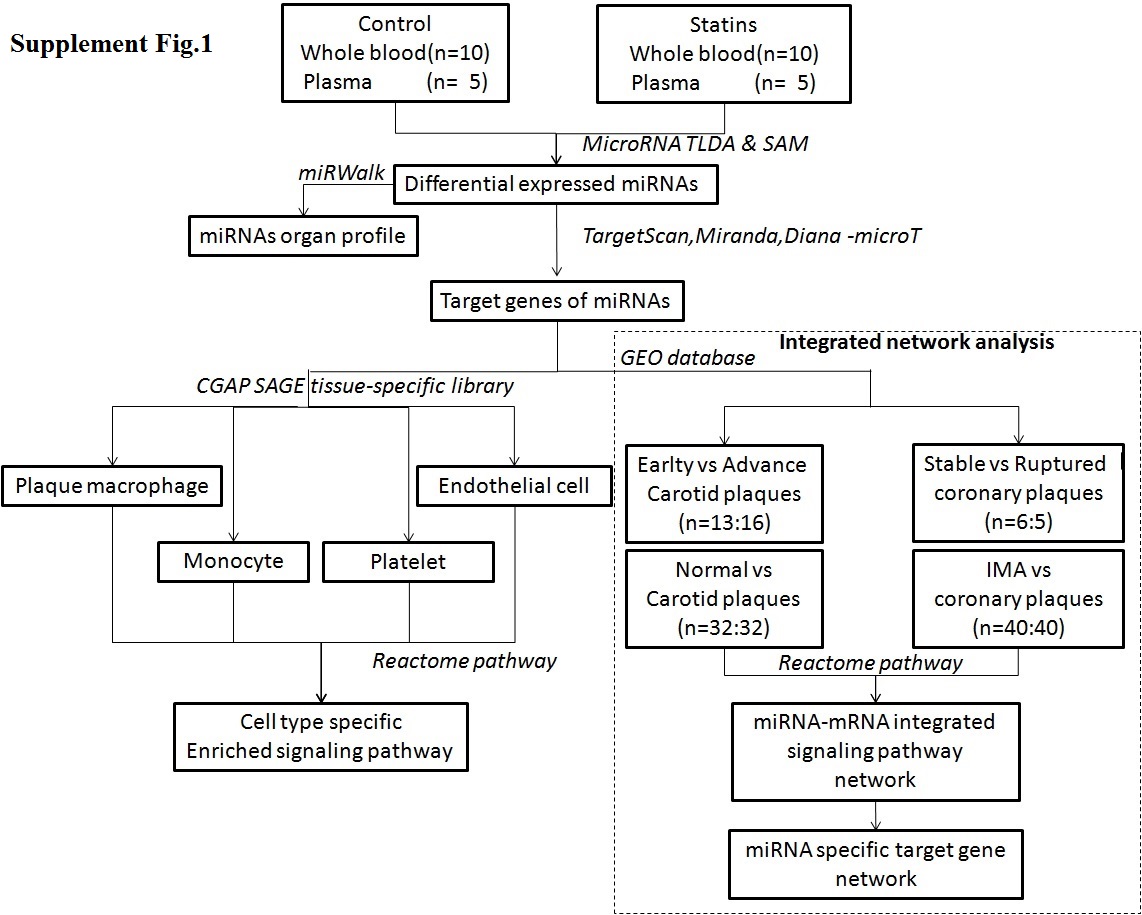

Supplement: Additional file 1: Figure S1. — Study work flow diagram. Whole blood samples were collected from UA patients (n = 30) enrolled at Peking University People’s Hospital. TLDA, TaqMan Low Density Arrays; SAM, Significance Analysis of Microarrays; CGAP SAGE, Cancer Genome Anatomy Project Serial Analysis of Gene Expression; GEO, Gene expression Omnibus. [file 12920_2015_82_MOESM1_ESM.jpeg]

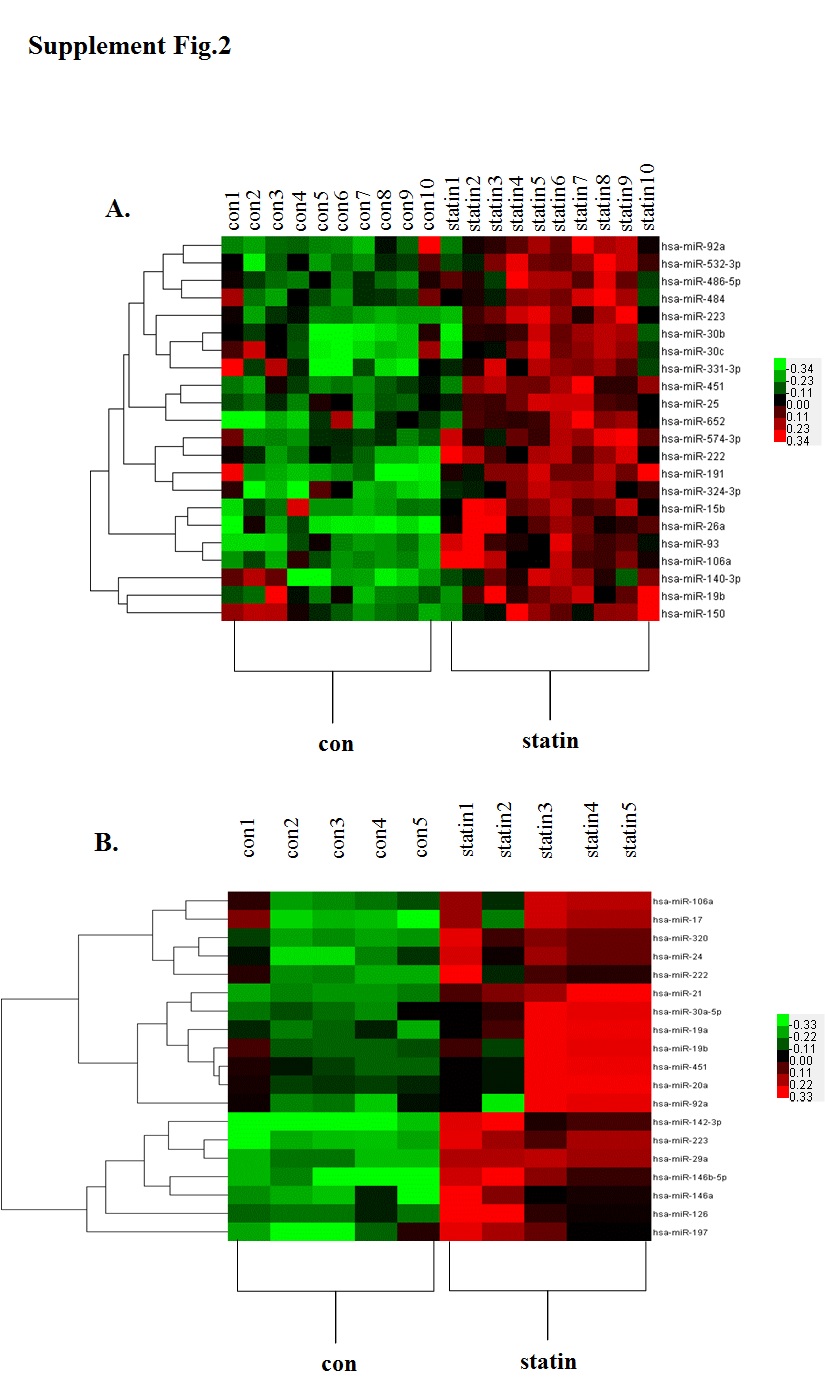

Supplement: Additional file 2: — Profile of circulating miRNAs in non-statin group and statin group. A: Whole blood differential miRNAs profiles. B: plasma miRNAs differential miRNAs profiles. Heatmap displays the levels of significantly differential expressed miRNAs. Color intensity represents the expression value in each row, bright red indicates the high expression level and the bright green indicates the low expression level. [file 12920_2015_82_MOESM2_ESM.jpeg]

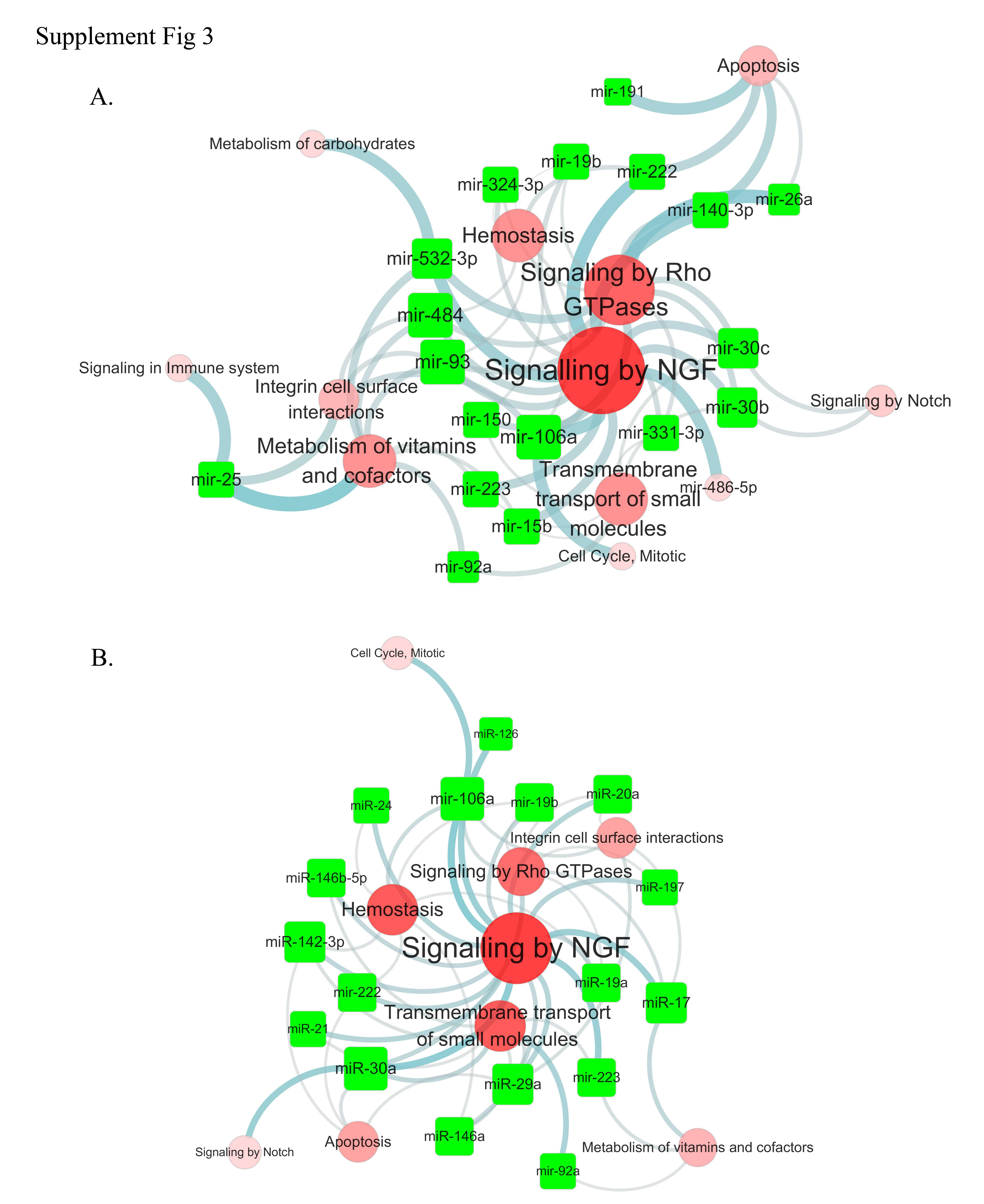

Supplement: Additional file 6: Figure S3. — MicroRNA target signaling pathway in the human cartoid plaque. The data which compared human carotid plaque and intact carotid tissue were obtained from GEO database. SAM was used to analyze the significantly differentially expressed genes (FDR < 0.05). Those genes enriched pathways and miRNA target gene pathways were clustered by DAVID based on Reactome pathway. A: Whole blood miRNAs target pathways. B: plasma miRNAs target pathways. Illustration combined the miRNA target pathways which were also involved in the coronary atherosclerosis plaque. [file 12920_2015_82_MOESM6_ESM.jpeg]

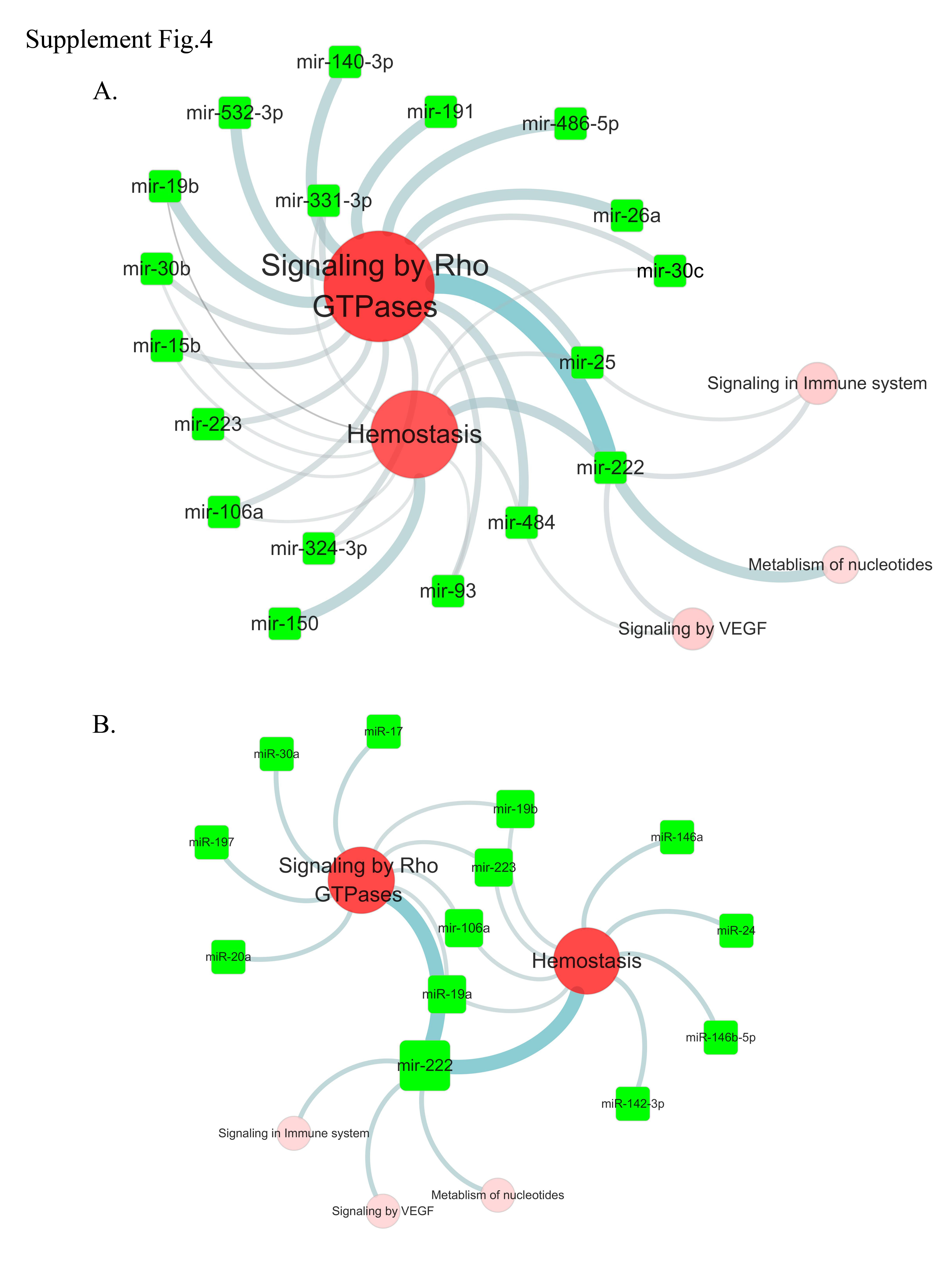

Supplement: Additional file 7: Figure S4. — MicroRNA target signaling pathway in the human advance carotid plaque. The data which compared advance carotid plaque and early carotid plaque were obtained from GEO database. SAM was used to analyze the significantly differentially expressed genes (FDR < 0.05). Those genes enriched pathways and miRNA target gene pathways were clustered by DAVID based on Reactome pathway. A: Whole blood miRNAs target pathways. B: plasma miRNAs target pathways. Illustration combined the miRNA target pathways which were also involved in the coronary atherosclerosis plaque. [file 12920_2015_82_MOESM7_ESM.jpeg]
