# Supplementary material for: Effects of statin on circulating microRNAome and predicted function regulatory network in patients with unstable angina
Source: BMC Med Genomics. 2015 Mar 13;8:12. doi: 10.1186/s12920-015-0082-4 (PMC4364658; doi:10.1186/s12920-015-0082-4)
Supplement: Additional file 3: Table S1. — MiRNAs organ expression profile were generate from miRWalk database. The organ expression profiles of the miRNAs were based on published data. [file 12920_2015_82_MOESM3_ESM.pdf]

	blood	monocyte	macrophage	platelet	endothelial cell
hsa-miR-191	hsa-miR-191		hsa-miR-191		
hsa-miR-92a	hsa-miR-92a	hsa-miR-92a	hsa-miR-92a		
hsa-miR-532-3p	hsa-miR-532-3p				
hsa-miR-574-3p					
hsa-miR-30b	hsa-miR-30b		hsa-miR-30b	hsa-miR-30b	
hsa-miR-486-5p	hsa-miR-486-5p				
hsa-miR-223	hsa-miR-223	hsa-miR-223	hsa-miR-223	hsa-miR-223	hsa-mir-223
hsa-miR-30c	hsa-miR-30c	hsa-miR-30c		hsa-miR-30c	
hsa-miR-484					
hsa-miR-451	hsa-miR-451	hsa-miR-451	hsa-miR-451	hsa-miR-451	
hsa-miR-331-3p	hsa-miR-331-3p				
hsa-miR-25	hsa-miR-25				
hsa-miR-222	hsa-miR-222	hsa-miR-222	hsa-miR-222		hsa-mir-222
hsa-miR-652					
hsa-miR-19b	hsa-miR-19b	hsa-miR-19b			
hsa-miR-15b	hsa-miR-15b	hsa-miR-15b	hsa-miR-15b	hsa-miR-15b	hsa-mir-15b
hsa-miR-140-3p	hsa-miR-140-3p				
hsa-miR-26a	hsa-miR-26a	hsa-miR-26a	hsa-miR-26a		
hsa-miR-324-3p					
hsa-miR-150	hsa-miR-150	hsa-miR-150	hsa-miR-150	hsa-miR-150	hsa-mir-150
hsa-miR-93	hsa-miR-93		hsa-miR-93		hsa-mir-93
hsa-miR-106a	hsa-miR-106a	hsa-miR-106a	hsa-miR-106a		hsa-mir-106a
hsa-miR-21	hsa-miR-21	hsa-miR-21	hsa-miR-21		hsa-miR-21
hsa-miR-17	hsa-miR-17		hsa-miR-17	hsa-miR-17	hsa-miR-17
hsa-miR-320					
hsa-miR-24	hsa-miR-24	hsa-miR-24	hsa-miR-24	hsa-miR-24	hsa-miR-24
hsa-miR-146b-5p	hsa-miR-146b-5p	hsa-miR-146b	hsa-miR-146b-	hsa-miR-146b	hsa-miR-146b
hsa-miR-142-3p	hsa-miR-142-3p	hsa-miR-142-	hsa-miR-142-3	hsa-miR-142-	hsa-miR-142-
hsa-miR-19a	hsa-miR-19a				hsa-miR-19a
hsa-miR-30a-5p					
hsa-miR-126	hsa-miR-126	hsa-miR-126	hsa-miR-126	hsa-miR-126	hsa-miR-126
hsa-miR-197	hsa-miR-197			hsa-miR-197	
hsa-miR-20a	hsa-miR-20a	hsa-miR-20a	hsa-miR-20a		hsa-miR-20a
hsa-miR-29a	hsa-miR-29a				
hsa-miR-146a	hsa-miR-146a	hsa-miR-146a	hsa-miR-146a	hsa-miR-146a	hsa-miR-146a
hsa-miR-222	hsa-miR-222	hsa-miR-222	hsa-miR-222		hsa-mir-222
hsa-miR-223	hsa-miR-223	hsa-miR-223	hsa-miR-223	hsa-miR-223	hsa-mir-223
hsa-miR-19b	hsa-miR-19b	hsa-miR-19b			
hsa-miR-92a	hsa-miR-92a	hsa-miR-92a	hsa-miR-92a		
hsa-miR-451	hsa-miR-451	hsa-miR-451	hsa-miR-451	hsa-miR-451	
hsa-miR-106a	hsa-miR-106a	hsa-miR-106a	hsa-miR-106a		hsa-mir-106a

−5p
3p
